# Centennial-scale solar forcing of the South American Monsoon System recorded in stalagmites

**DOI:** 10.1038/srep24762

**Published:** 2016-04-21

**Authors:** Valdir F. Novello, Mathias Vuille, Francisco W. Cruz, Nicolás M. Stríkis, Marcos Saito de Paula, R. Lawrence Edwards, Hai Cheng, Ivo Karmann, Plínio F. Jaqueto, Ricardo I. F. Trindade, Gelvam A. Hartmann, Jean S. Moquet

**Affiliations:** 1 Instituto de Geociências, Universidade de São Paulo, São Paulo 05508-090, Brazil; 2 Department of Atmospheric and Environmental Sciences, University at Albany, Albany, New York 12222, USA; 3 Departamento de Geoquímica, Universidade Federal Fluminense, Niterói, Rio de Janeiro 24220-900, Brazil; 4 Department of Earth Sciences, University of Minnesota, Minneapolis, Minnesota 55455, USA; 5 Institute of Global Environmental Change, Xi’an Jiaotong University, Xi’an 710049, China; 6 Instituto de Astronomia, Geofísica e Ciências Atmosféricas, Universidade de São Paulo, São Paulo, Brazil; 7 Observatório Nacional, Rio de Janeiro 20921-400, Brazil

## Abstract

The South American Monsoon System (SAMS) is generally considered to be highly sensitive to Northern Hemisphere (NH) temperature variations on multi-centennial timescales. The direct influence of solar forcing on moisture convergence in global monsoon systems on the other hand, while well explored in modeling studies, has hitherto not been documented in proxy data from the SAMS region. Hence little is known about the sensitivity of the SAMS to solar forcing over the past millennium and how it might compete or constructively interfere with NH temperature variations that occurred primarily in response to volcanic forcing. Here we present a new annually-resolved oxygen isotope record from a 1500-year long stalagmite recording past changes in precipitation in the hitherto unsampled core region of the SAMS. This record details how solar variability consistently modulated the strength of the SAMS on centennial time scales during the past 1500 years. Solar forcing, besides the previously recognized influence from NH temperature changes and associated Intertropical Convergence Zone (ITCZ) shifts, appears as a major driver affecting SAMS intensity at centennial time scales.

Several studies have shown that monsoon systems and other large-scale convergence zones around the world are influenced by solar variability^1^,^2^,^3^,^4^,^5^,^6^,^7^,^8^,^9^,^10^,^11^,^12^. Meehl *et al.*^12^, for example, reported that peaks in solar forcing increase the energy input to the surface ocean at subtropical latitudes, thereby enhancing evaporation and near-surface moisture, which is carried by the trade winds to the convergence zones. Through this mechanism convective activity in the regions influenced by the upward branches of Hadley and Walker cells can be intensified, resulting in strengthened regional tropical precipitation regimes due to enhanced solar forcing^8^. This process is reinforced by several positive feedbacks involving amplification of solar forcing by coupled air-sea dynamics, cloud formation and stratospheric warming due to enhanced UV absorption through increased stratospheric ozone concentration^11^,^12^. Model simulations support the idea that the upper-tropospheric subtropical jet is shifted poleward, accompanying the southward expansion of the southern branch of the Hadley cell, following the stratospheric ozone increase and warming during solar irradiance maxima^13^,^14^,^15^,^16^. The resulting adjustment in the Hadley cell modulates the position of the Intertropical Convergence Zone (ITCZ)^9^, leading to a southward displacement of this system during periods of increased solar irradiance. Since a more southerly position of the ITCZ enhances moisture convergence over the Amazon basin^17^,^18^, a more southerly position of the Hadley Cell will lead to a strengthening of the SAMS. So far, however, this mechanism has not been given much attention over the SAMS domain as Northern Hemisphere (NH) temperature is generally considered the main driver associated with SAMS variability and shifts in the latitudinal position of the ITCZ during the last two millennia. Several studies, for example, have tied changes in the mean state of the SAMS during anomalous climate periods such as the Little Ice Age (LIA) and Medieval Climate Anomaly (MCA) to this latter mecahnism^18^,^19^.

The NH temperature is of course also somewhat dependent on changes in solar irradiance, with stronger warming in the NH during periods of increased solar irradiance, thereby contributing to a northward shift of the ITCZ and a weakening of the SAMS (and vice-versa during periods of reduced solar irradiance)^18^,^19^. However, most of the NH temperature excursions over the past millennium, including the LIA cooling, occurred first and foremost in response to volcanic forcing and were only to a very small extent influenced by solar variability^20^. In this sense, the NH warming/cooling and the direct solar irradiance influence on tropical convergence zones can be considered two separate forcings, which at times may oppose or constructively interfere with one another in modulating the SAMS intensity.

To document past changes in SAMS intensity and its sensitivity to solar radiative forcing, a record near the core region of SAMS activity is required. Here, we present a new high-resolution paleoclimate record based on δ^18^O data from stalagmites collected in the core region of the SAMS (Brazil), in the northern portion of the La Plata Basin, along the transition zone with the Amazon Basin. The record is developed from two stalagmites (ALHO6 and CUR4) collected from Pau d’Alho and Curupira caves respectively, and is representative of SAMS variability in its active core, covering the last 1500 years with accurate geochronology based on U-Th ages and annual layer counting.

## Study Site, Samples and Environmental Parameters

### Cave location

**Figure 1:**
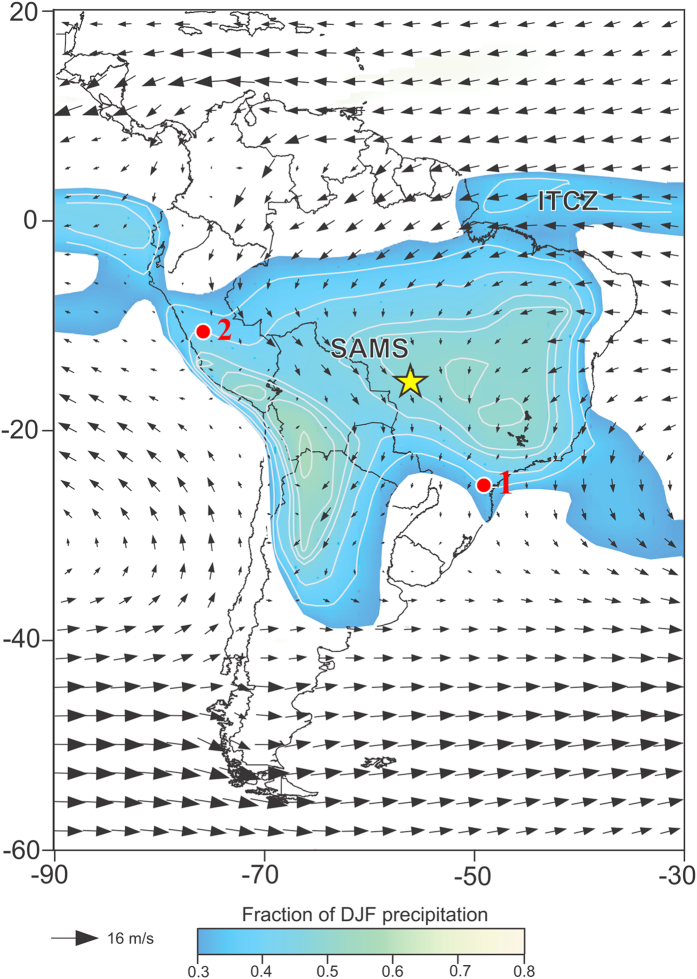
Map of South America with location of paleoclimate records discussed in text, austral summer (Dec.–Feb, DJF) 850 hPa wind field and fractional DJF precipitation. Color shading indicates regions where fraction of total annual precipitation falling during austral summer (DJF) > 0.3, which is congruent with the extent of the SAMS over the continent; contour interval is 0.05. Wind data is from ERA-Interim^38^ and precipitation data from GPCC^39^, with averages calculated over period 1979–2014. Yellow star indicates our speleothem site. Other sites include 1- Speleothem record from Cristal cave (stalagmite CR1)^18^, 2- Laguna Pumacocha^19^. The figure was created using the software Adobe Illustrator CS6 version 16.0.0.

This study is based on two stalagmites, CUR4 (Supplementary Fig. S1) collected in the Curupira cave (15°12′01″S, 56°47′02″W) and ALHO6 (Supplementary Fig. S1) collected in the Pau d’Alho cave (15°12′20″S, 56°48′41″W). The two caves are within ~3 km distance from each other and are located near Rosário Oeste City (Fig. 1) in Mato Grosso State, Brazil.

### Samples

The ALHO6 sample is a 24 cm long calcite stalagmite, which grew continuously between ~490 to 1860 years A.D. Its isotopic profile consists of 1185 δ^18^O samples with the geochronology established from 12 U-Th dates (Supplementary Table S1) and annual layer counting. The modern portion is complemented with the aragonite stalagmite CUR4 that was sampled between 1795 and 1970 (top) A.D. with 254 δ^18^O samples and 4 U-Th dates. The stalagmites are characterized by an offset in their δ^18^O values of approximately 1% during the period of overlap, reflecting the different equilibrium fractionation conditions between calcite and aragonite^21^. The mean growth rate of ALHO6 and CUR4 is 0.17 mm/yr and 0.20 mm/yr, respectively, yielding an average δ^18^O resolution of 1.1 and 0.7 years per sample, respectively.

### Climate

The climate in Rosário Oeste City is tropical, with a four to five months long dry season. The mean annual temperature is 25.5 °C and the annual mean precipitation is 1440 mm, with 90% of this amount falling during the monsoon season, October to April (results based on data set collected between 1968 and 2013 from meteorological stations located in Rosário Oeste City, data from www.ana.gov). Nearly all moisture originates from evapotranspiration over the Amazon forest, characterizing this site as being sourced almost exclusively by precipitation from the SAMS^22^.

### Cave Monitoring

The monitoring performed during a 13 month period in Pau d’Alho cave shows that the air in the hall where the sample ALHO6 was collected was constantly saturated, while the relative humidity at the cave entrance was 33% during the dry months (August-September) and around 90% during the wet season (Supplementary Fig. S2). The temperature was monitored over a period of two years and in the hall showed a minimum of 25.5 ± 0.1 °C and a maximum of 26.0 ± 0.1 °C, while at the entrance of this cave the temperature varied between 16.0 ± 0.1 °C and 26.8 ± 0.1 °C (Supplementary Fig. S3). In Curupira cave, the temperature was monitored during 10 months (Supplementary Fig. S4) and the values in the hall where the stalagmite CUR4 was collected were consistently at 25.7 ± 0.1 °C during all of the monitored period, with the relative humidity at a constant 100% during the entire monitoring period. Both caves are characterized by high CO_2_ concentrations in the halls where the stalagmites were collected (>1000 ppm in Pau d’Alho cave and >500 ppm in Curupira cave), as evidenced by repeated measurements, listed in Supplementary Table S2. These measurements indicate that both caves present excellent conditions for carbonate to precipitate close to isotopic equilibrium.

### Amount effect

In tropical and subtropical areas of South America the “amount effect” on interannual timescales leads to more depleted δ^18^O values in rainfall in anomalously wet years and relatively more enriched δ^18^O values during drier years. This relationship was documented in observations and model simulations by Vuille *et al.*^23^ and subsequently applied in several paleoclimate reconstructions based on speleothems at locations where the majority of their precipitation is due to the SAMS^18^,^24^,^25^,^26^,^27^. Observational data from the Global Network of Isotopes in Precipitation program of the International Atomic Energy Agency (GNIP-IAEA) confirm the negative correlation on interannual time scales between monthly anomalies of precipitation and monthly δ^18^O anomalies at a station in Cuiabá city (Supplementary Fig. S5), located ~85 km from the caves considered in this study. In addition, the δ^18^O and δD values of rainwater from the GNIP program in Cuiabá, as well as the δ^18^O and δD values of rainwater and cave drip water collected by the authors during both dry and wet seasons in Pau d’Alho and Curupira caves, all plot on top of the global meteoric water line (GMWL) (Supplementary Fig. S6). This behavior indicates that the isotopic composition of cave drip water can be traced back to the isotopic composition of rainfall, further corroborating the potential of speleothem oxygen isotope records to reconstruct precipitation (Supplementary Fig. S6). It is further important to note that sites downstream from the core monsoon region receive moisture, which is depleted in heavy isotopes, depending on the degree of rainout upstream^18^. Hence the isotopic composition of rainfall at these sites reflects the strength of monsoon precipitation, even in the absence of a strong local amount effect.

## Results and Discussion

**Figure 2:**
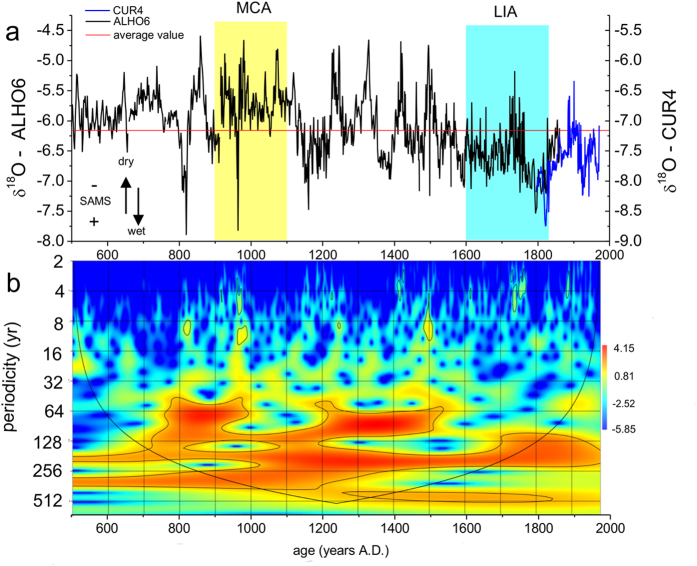
Time series and wavelet analysis of stalagmite δ^18^O record from Mato Grosso State (Brazil). (**a**) ALHO6 (black) and CUR4 (blue) stalagmite δ^18^O (VPDB) records from Mato Grosso State, Brazil. The scale for CUR4 (right y-axis) is shifted by 1 per mil relative to the ALHO6 scale to account for the different equilibrium fractionation conditions between calcite and aragonite. The yellow shading highlights the relatively dry MCA interval, while the blue shading indicates the wetter LIA period. (**b**) Wavelet analysis performed with ALHO6+CUR4 δ^18^O signal using the software PAST^40^ and the Morlet mother wavelet. Black lines indicate the 95% significance level and the cone of influence (region over which record length is sufficient to interpret results).

The spectral analysis of the δ^18^O values of stalagmites ALHO6 and CUR4 (Fig. 2), performed with the REDFIT method, reveals a significant periodicity at 208 years and marginally significant periodicities at 83, 31, 18–16, 11, 9 and 7–3 years (Fig. 2, Supplementary Fig. S7). The periodicities of 208, 83 and 11 years in the spectral analysis are close to the solar cycles of de Vries-Suess^28^, Gleissberg^29^ and the Schwabe^30^ sunspot cycle, respectively. The wavelet analysis of the ALHO6+CUR4 record (Fig. 2b) indicates that the main 208-year cycle is very robust and persists over the entire 1500 years. This same periodicity (210 year) is also highlighted in an independent spectral analysis performed by using the Lomb periodogram method (Supplementary Fig. S8). The 83 year cycle is stronger between ~750–1000 AD and ~1200–1500 AD, while the 11 year cycle appears more randomly distributed over the record. The periodicities of 31 and 18–16 years do not emerge as significant in the wavelet analysis, but can be related to the influence of the Pacific Decadal Oscillation (PDO) in the region^31^. The periodicities of 7–3 years are most likely related to ENSO variability^31^. In addition, the cross-wavelet analysis between ALHO6 and total solar irradiance^32^ shows a clear correlation at a periodicity of approximately 208 years. The solar irradiance is in clear anti-phase with the δ^18^O variability of the stalagmite (Supplementary Fig. S9), although a slight phase discrepancy is apparent before 1100 AD, with the solar forcing leading the δ^18^O response. The exact reason for this phase delay is not clear but may be related to dating uncertainties or delays and feedbacks in the climate system. Both δ^18^O signal and the solar irradiance also show strong power on the multidecadal timescale (83 years), (Supplementary Fig. S9), yet the cross-wavelet analysis indicates that the phasing between the two records oscillates. This suggest a less consistent influence of solar irradiance on the SAMS on multidecadal time scales, likely modulated by other oceanic and atmospheric processes. Below we therefore focus on the most pervasive and coherent response to solar irradiance changes, which is on the 208-year time scale.

**Figure 3:**
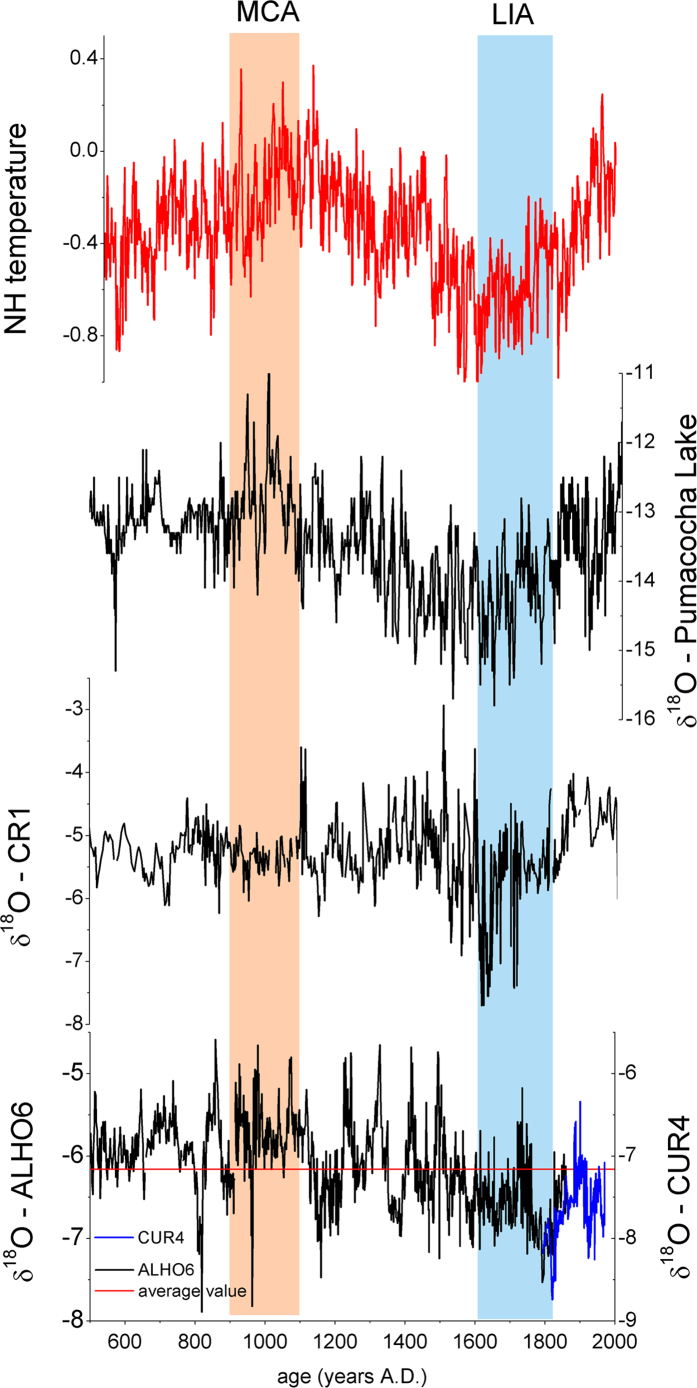
Comparison between the reconstruction of NH temperature^41^ with the δ^18^O records from Laguna Pumacocha^19^ located in the Peruvian Andes, CR1 stalagmite^18^ located in SE Brazil and our ALHO6+CUR4 record.

The Medieval Climate Anomaly (MCA) and Little Ice Age (LIA), have been documented as periods with significant changes in the mean state of the SAMS in δ^18^O records from the Andes^19^,^24^ and SE Brazil^18^,. This is consistent with our new record (Fig. 3), which also shows significant departures from the mean state during both the MCA (900–1100 AD) and the LIA (1600–1820 AD), with both periods defined as in Vuille *et al.*^18^. The δ^18^O value during the MCA is −5.71 permil, while it drops to −6.57 permil during the LIA (Fig. 2a). The long-term mean value for the past millennium (850–1850 AD, with MCA and LIA excluded) is −6.18 permil. A two-tailed student’s t-test confirms that the mean the δ^18^O value, and hence the mean state of the SAMS, is significantly different from the long-term mean during both the MCA and the LIA (p < 0.001). Clearly our record confirms previous reports of significant mean state changes in SAMS intensity and is consistent with interpretations that have attributed these changes to NH temperature anomalies and related shifts in the Atlantic ITCZ^18^,^19^ (Fig. 3).

Aside from these mean-state changes, however, our record shows a pervasive 208-year periodicity throughout the entire record, which is difficult to reconcile with NH temperature variability, but consistent with changes in solar irradiance. The absolute magnitude of the solar forcing is weak and not sufficient to directly affect the SAMS. But through the feedbacks^10^ discussed above, changes in total solar irradiance may lead to a strengthening of the SAMS and increased rainfall in the tropics, including over the ITCZ and SAMS domains.

**Figure 4:**
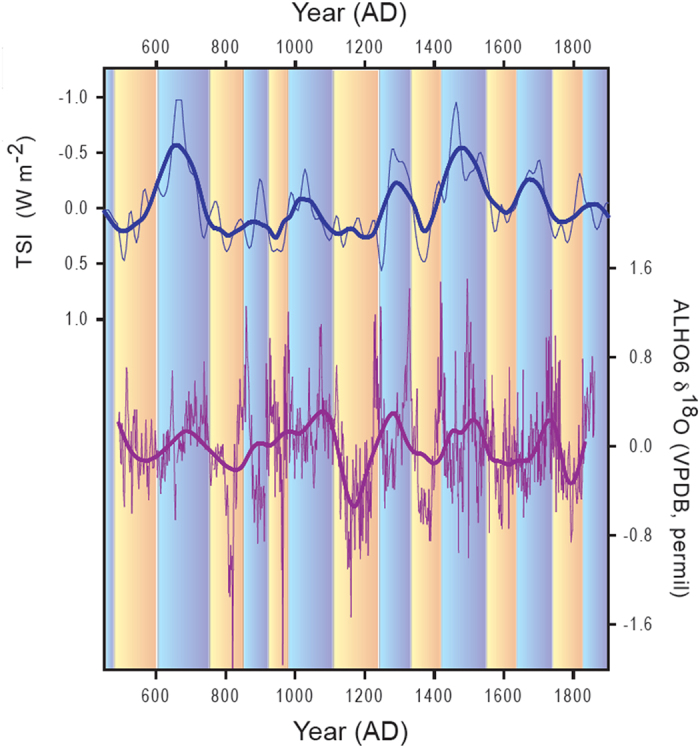
Comparison between anomalies in total solar irradiance^32^ (TSI, in W m^−2^, blue) and our ALHO6 δ^18^ O record (in permil, purple). Thin lines show raw anomalies; thick lines are low-pass filtered with a nearest neighbor 208-yr window using a Gaussian kernel. ALHO6 was detrended with a 3^rd^ order polynomial function prior to filtering to remove long-term trends associated with mean state changes from Medieval Climate Anomaly (MCA) to Little Ice Age (LIA). Orange (blue) shading highlights periods of above (below) average low-pass filtered TSI respectively, which tend to coincide with decreased (increased) δ^18^O.

A simple composite analysis demonstrates that the most negative values in the detrended ALHO6 record are indeed associated with periods of high solar irradiance and vice versa (Fig. 4). The most negative anomalies (δ^18^O <−0.5) coincide with solar irradiance anomalies that average +0.27 W m^−2^, significantly higher (p < 0.001, two-tailed students t-test) than the solar irradiance anomalies (−0.07 W m^−2^) during periods when δ^18^O anomalies are most enriched in the heavy isotope (δ^18^O anomalies >0.5). Note that this threshold of δ^18^O anomalies >|0.5|, while somewhat subjective, serves as a reasonable delineation of the extremes in this record as it coincides roughly with the 10- and 90-percentile of the δ^18^O distribution.

The SAMS is of course also influenced by a number of other forcings. For example, multidecadal variability associated with the Atlantic Multidecadal Oscillation (AMO) is well known to affect the SAMS^24^,^26^,^33^, with a more southerly position of the ITCZ during its negative phase. Indeed the 83 yr periodicity, which shows up in our record between 750 and 1000 A.D. and then again between 1200 and 1500 A.D. (Fig. 2b), may well be associated with the AMO. Other potential forcing mechanisms capable of modulating the SAMS involve links with the tropical Pacific and the El Niño – Southern Oscillation (ENSO), which is the dominant mode influencing South American climate on interannual time scales^31^ and also affects precipitation over the SAMS region. Over tropical South America, the eastward shift of the Walker circulation leads to a weakening of the SAMS during El Niño^34^ and below average precipitation in NE Brazil, the northern Amazon basin and the tropical Andes from 10°N to 20°S. At the same time the strengthening of the Andean low level jet^35^,^36^ increases the moisture flux toward the subtropical plains and SE Brazil. Despite this strong influence of ENSO on South American climate, however, a centennial-scale influence of ENSO is rather unlikely. As shown by Vuille *et al.*^18^, southeastern Brazil, which sees a significant increase in precipitation during El Niño and the tropical Andes, which tend to experience dry conditions at that time, show a coherent response in centennial-scale monsoon strength over the past two millennia, which is inconsistent with ENSO forcing. In addition, our study site is located in between the two poles affected by increased precipitation during El Niño (subtropical plains to the south) and the regions seeing reduced precipitation (northern Amazon and much of tropical South America). Indeed, studies show that the location of our cave site is rather insensitive to the ENSO phase and does not experience significant rainfall departures during either El Niño or La Niña^31^. Notwithstanding, it is possible of course that these mechanisms may have changed somewhat over time, explaining the spectral peaks around 3–7 years in our record (Supplementary Fig. S7).

It is noteworthy that Bird *et al.*^19^ also associated observed mean state changes in the SAMS between the MCA and LIA, derived from δ^18^O in lake calcite in the Andes of Peru, with anomalous radiative forcing, at least partly driven by changes in total solar irradiance. In their interpretation, also discussed in Vuille *et al.*^18^, the main focus was on how changes in radiative forcing influence NH temperature. It is well known that reductions in radiative forcing and the resulting cooling of the NH lead to a southward displacement of the ITCZ, thereby strengthening the SAMS^18^. Our record, however, suggests that on centennial time scales the direct influence of solar forcing on tropical moisture convergence may have the opposite effect and actually lead to a strengthening of the SAMS. More recent studies, clearly document that the solar forcing was insufficient to induce the observed NH cooling during the LIA^20^. Instead, NH aerosol forcing related to volcanic eruptions has emerged as the main factor leading to the hemispheric temperature asymmetry, resulting in the southward shift of the ITCZ at that time. Our record also documents this intensification of the SAMS during the LIA, consistent with previous records (Fig. 3). In addition, however this new monsoon record also shows a pervasive solar signal at the 208-year frequency, which has not previously been documented in the region (Figs 2 and 4). The solar signal is characterized by a clear periodicity of 208 years and hence very different in character from the individual climate anomalies during the MCA and LIA. Our record indicates that on centennial time scales the periodic solar forcing leads to a strong modulation of the SAMS, with enhanced SAMS intensity during periods of strong solar forcing. This solar forcing is superimposed on the mean state changes brought on by NH temperature anomalies due to hemispherically asymmetric (i.e. volcanic) forcing. Indeed, model simulations^5^ suggest that on centennial time scales the global monsoon strength may respond more directly to the effective solar forcing. This idea is supported by our record, which shows strong variability on centennial time scales related to solar irradiance, superimposed on its response to changes in hemispheric temperature gradients as seen during the LIA. It is also consistent with the fact that the phase relationship between solar forcing and our δ^18^O speleothem record is stable only on the 208 yr time scale, but varies throughout the record at other frequencies (Supplementary Fig. S9).

## Conclusion

The main focus of this study was to document, for the first time, a pervasive, 208-year periodicity of the South American monsoon, which persists throughout the past 1500 years. This periodicity is apparent in our new high-resolution speleothem record from south-central Brazil, located in the core of the SAMS. The documented coherent in-phase relationship on centennial timescales between the precipitation in our speleothem record and a reconstruction of total solar irradiance is consistent with model simulations, which indicate that tropical precipitation is sensitive to solar forcing^10^,^12^ and that the southern hemisphere monsoon in particular responded in a sensitive way to solar forcing during the past millennium^7^. Is intriguing that this strong solar signal at the 208-year time scale was not identified in previous publications presenting other monsoon records from South America, with exception of a record from northeast Brazil^26^. Most previous studies from the region have instead focused on the much more obvious mean state changes during the LIA and MCA. While these δ^18^O excursions are also evident in our record, they are not quite as strong, suggesting that these events may be more prominently recorded at more distal monsoon sites. To what extent the solar signal affected the entire monsoon region or whether its influence was limited to the core monsoon region over the southern Amazon basin will require additional records, but also a thorough reanalysis of some of the existing records.

## Methods

### U-Th dating method and δ^18^O analysis

The stalagmites were dated by U-Th method at the Minnesota Isotope Laboratory (USA), using a multi-collector inductively coupled plasma mass spectrometry technique (MC-ICP-MS, Thermo-Finnigan NEPTUNE), according to the procedures described in Cheng *et al.*^37^. Oxygen isotope ratios are expressed in δ notation, the per mil deviation from the Vienna Peedee Belemnite (VPDB) standard. δ^18^O = [((^18^O/^16^O) sample/(^18^O/^16^O)VPDB) − 1] × 1000. For each measurement, approximately 100 μg of powder was drilled from the sample and analyzed with an on-line, automated, carbonate preparation system linked to a Thermo-Finnigan Delta Plus Advantage at the Stable Isotope Laboratory of the Geosciences Institute of Universidade de São Paulo (LIESP-CPGeo-IGc-USP). The speleothem reproducibility of standard materials is approximately 0.1% for δ^18^O.

### Geochronology

The ALHO6 stalagmite contains annual layers throughout its entire length, as confirmed by the good correspondence between layer counting and U/Th dates along the entire record. U/Th dates were therefore extrapolated to every single annual layer of ALHO6 stalagmite using a polynomial fit between the U/Th data and distance in mm of each stratigraphic layer counted from the stalagmite top. The polynomial fit exhibits an r^2^ = 0.99 and results in a polynomial geochronologic model of the δ^18^O data, assigning an absolute age to every individual stratigraphic layer (Supplementary Fig. S10). For the top portion of the stalagmite, located above the last obtained U/Th age, each stratigraphic layer was considered as one year. The comparison between the polynomial model (U-Th data and layer counting) with a model based only on the linear interpolation between the U-Th ages is shown in Supplementary Fig. S11. The CUR4 stalagmite did not present any visible stratigraphic layers; hence the geochronology from this sample was based entirely on a linear interpolation between the U-Th ages. For counting the annual laminae, a polished section of petrographic slabs made from stalagmite ALHO6 was photographed with a CCD camera to a stereo-microscope with transmitted light. The individual layers were counted with their respective thickness measured in the images using the software Corel Draw.

## Additional Information

**How to cite this article**: Novello, V. F. *et al.* Centennial-scale solar forcing of the South American Monsoon System recorded in stalagmites. *Sci. Rep.* **6**, 24762; doi: 10.1038/srep24762 (2016).

## Supplementary Material

Supplementary Information
