# Supplementary material for: Centennial-scale solar forcing of the South American Monsoon System recorded in stalagmites
Source: Sci Rep. 2016 Apr 21;6:24762. doi: 10.1038/srep24762 (PMC4838851; doi:10.1038/srep24762)
Supplement: Supplementary Information [file srep24762-s1.pdf]

Supplementary Information for

Centennial-scale solar forcing of the South American Monsoon System recorded in stalagmites

Valdir F. Novello[1*], Mathias Vuille[2], Francisco W. Cruz[1], Nicolás M. Stríkis[3], Marcos S. de Paula[1], R. Lawrence Edwards[4], Hai Cheng[5,4], Ivo Karmann[1], Plínio F. Jaqueto[6], Ricardo I. F. Trindade[6], Gelvam A. Hartmann[7], Jean S. Moquet[1]

[1]Instituto de Geociências, Universidade de São Paulo, São Paulo 05508-090, Brazil. [2]Department of Atmospheric and Environmental Sciences, University at Albany, Albany, New York 12222, USA. [3]Departamento de Geoquímica, Universidade Federal Fluminense, Niterói, Rio de Janeiro 24220-900. [4]Department of Earth Sciences, University of Minnesota, Minneapolis, Minnesota 55455, USA. [5]Institute of Global Environmental Change, Xi'an Jiaotong University, Xi'an 710049, China. [6]Instituto de Astronomia, Geofísica e Ciências Atmosféricas, Universidade de São Paulo, São Paulo, Brazil. [7]Observatório Nacional, Rio de Janeiro 20921-400, Brazil.

This document includes:

Figures S1 to S11

Tables 1 to 2

References

*To whom correspondence should be addressed.

e-mail: vfnovello@gmail.com

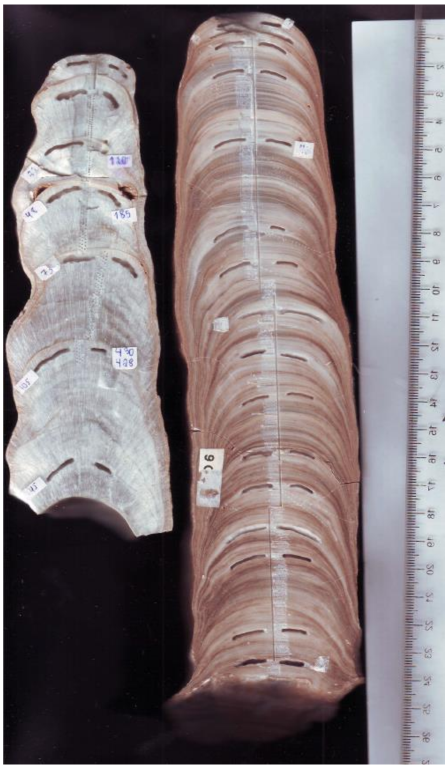

Supplementary Figure S1 | Stalagmites CUR4 (left) and ALHO6 (right).

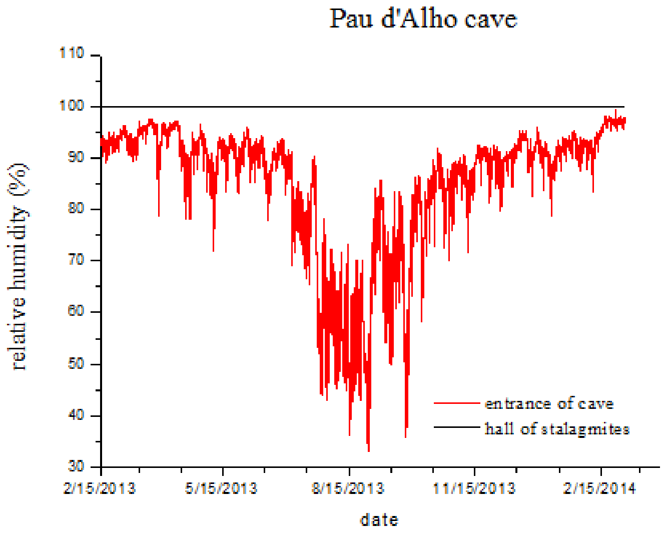

Supplementary Figure S2 | Relative humidity in the hall where the stalagmite ALHO6 was collected (black) and at the entrance (red) of the cave Pau d'Alho. The relative humidity was measured by HOBO data logger model U23.

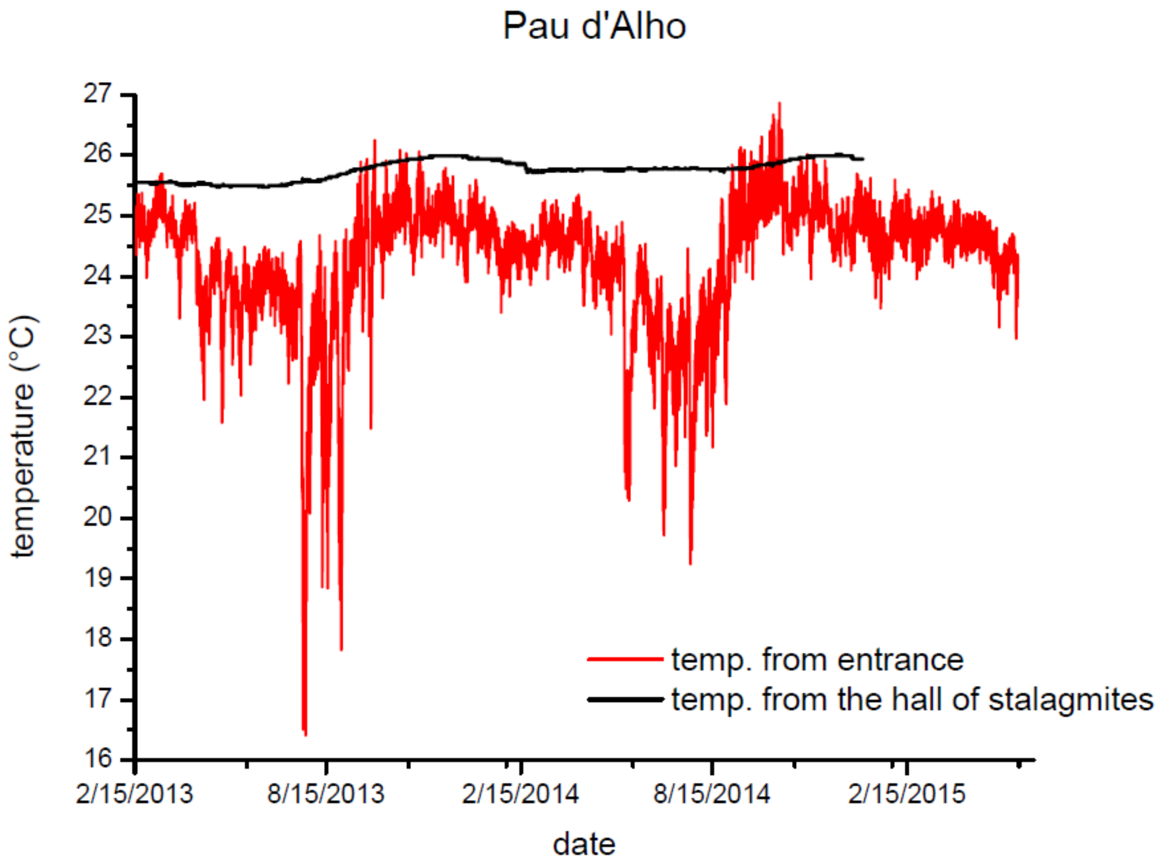

Supplementary Figure S3 | Temperature in the hall where the stalagmite ALHO6 was collected (black) and at the entrance (red) of the cave Pau d'Alho. The temperature was measured by HOBO data logger model U23.

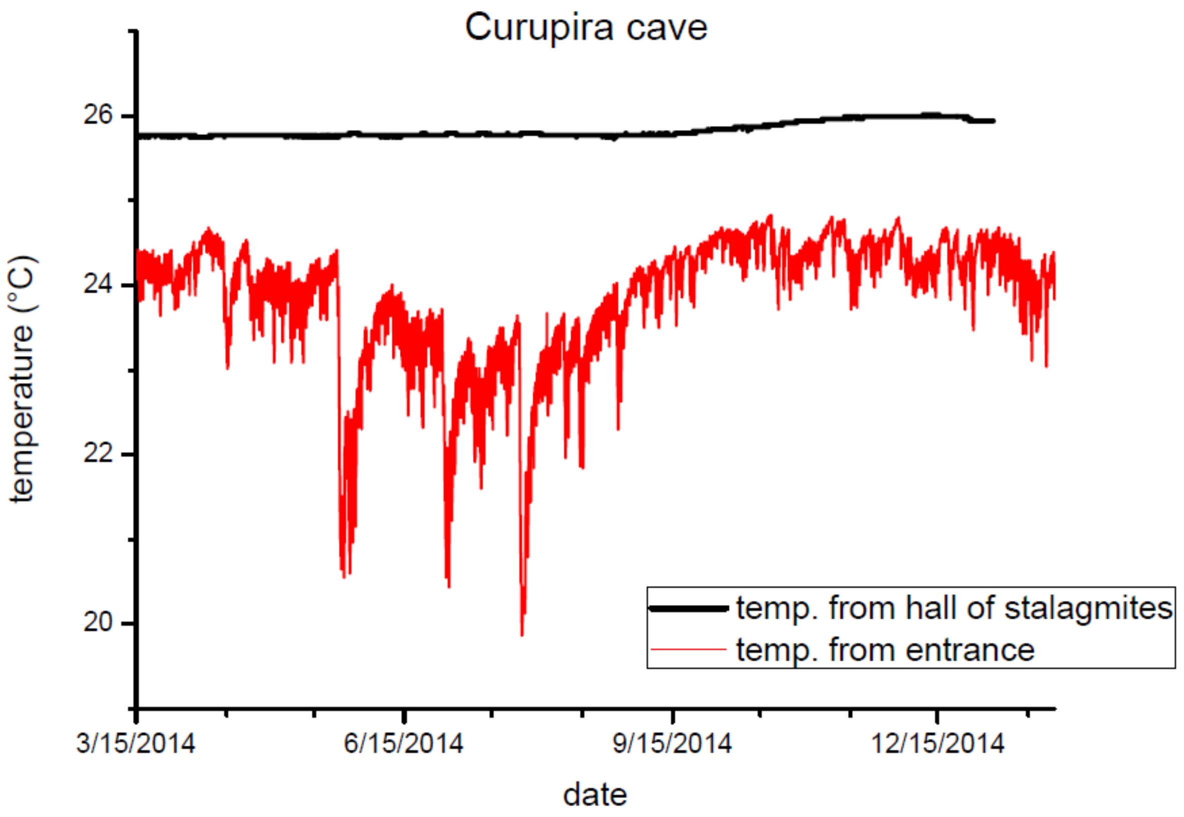

Supplementary Figure S4 | Temperature in the hall where the stalagmite CUR4 was collected (black) and at the entrance (red) of the cave Curupira. The temperature was measured by HOBO data logger model U23.

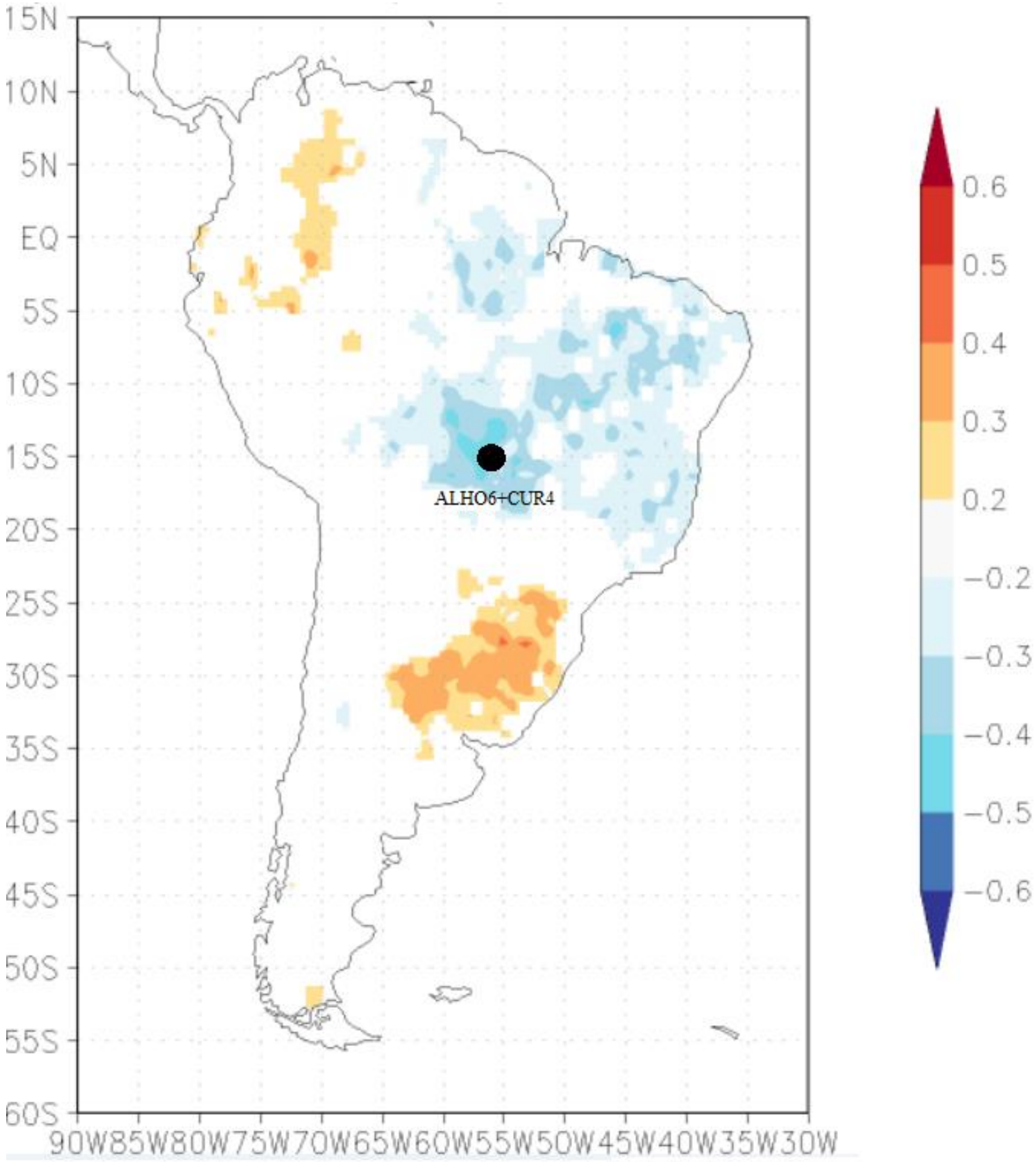

Supplementary Figure S5 | Spatial correlation between monsoon season (Nov-March) Cuiaba (located ~85 km from the caves considered in this study) monthly $\delta^{18}O$ anomalies and precipitation anomalies over South America between 1961 and 1987. Precipitation data is from GPCC V7[1] and $\delta^{18}O$ data is from IAEA-GNIP (available at http://www-naweb.iaea.org/napc/ih/IHS_resources_gnip.html). Shading indicates regions where correlations are significant at $p<0.1$. Figure created with Climate Explorer software available at: https://climexp.knmi.nl/start.cgi?id=someone@somewhere.

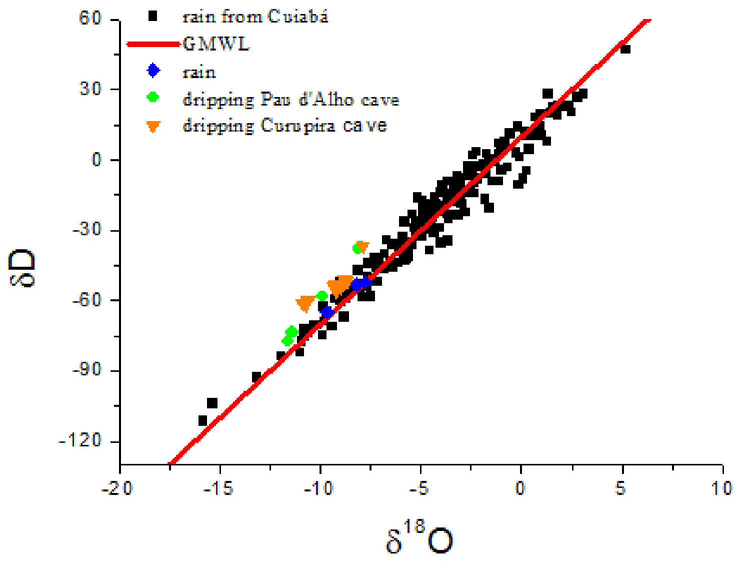

Supplementary Figure S6 | Relationship between $\delta^{18}O$ (VSMOV) and δD (VSMOV). The Global Meteoric Water Line (red line) is shown together with isotopic values from the IAEA-GNIP station (available at http://www-naweb.iaea.org/napc/ih/IHS_resources_gnip.html) located in Cuiabá city (black), rainwater isotopic values collected close to the caves (blue), drip water from the Pau d'Alho cave (orange) and drip water from the Curupira cave (green).

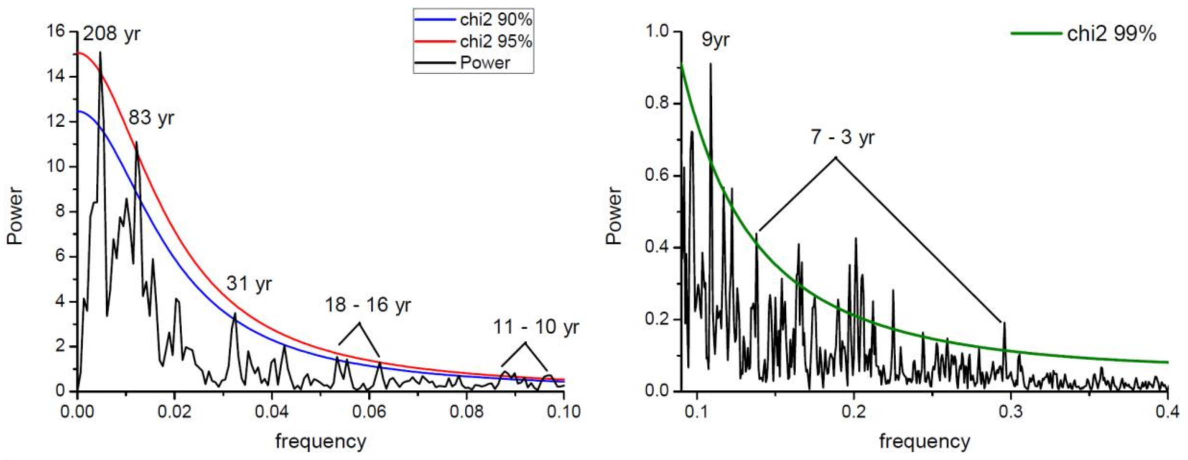

Supplementary Figure S7 | Spectral analysis (REDFIT) of our $\delta^{18}O$ stalagmite record performed with the software PAST[2]. The utilized parameters were: window: rectangle; oversample: 2; segment: 3. For the better visualization the results are shown separately for low frequencies at decadal to centennial scales (left) and high frequencies on interannual time scales (right). The blue, red and green lines represents the 90%, 95% and 99% of the chi-squared confidence level, respectively.

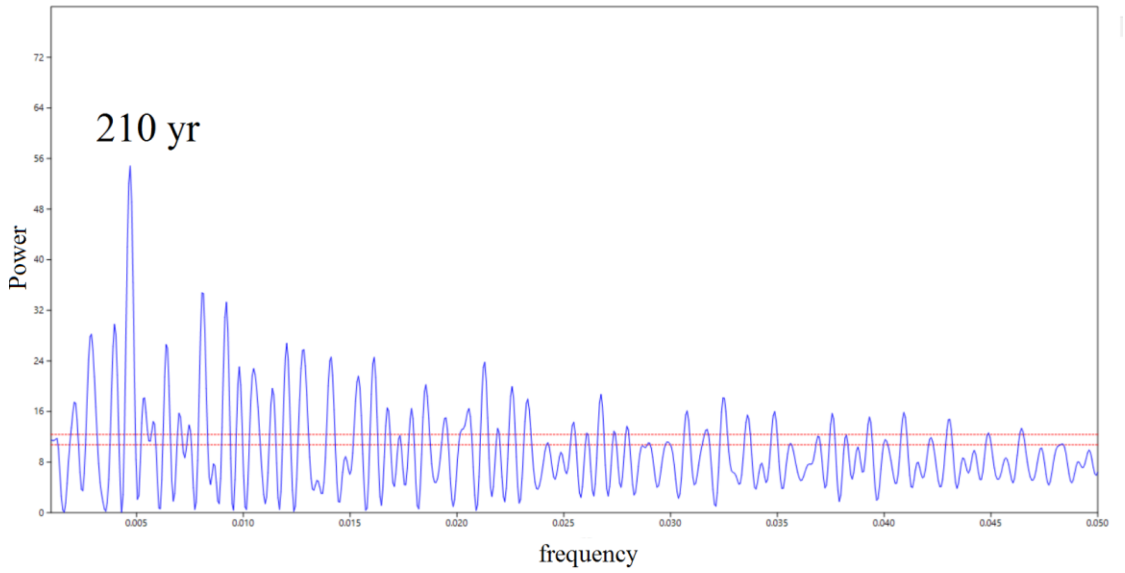

Supplementary Figure S8 | Spectral analysis (Lomb periodogram) of our $\delta^{18}O$ stalagmite record performed with the software PAST[2]. The two red lines indicate the 0.001- and 0.005-significance level, respectively.

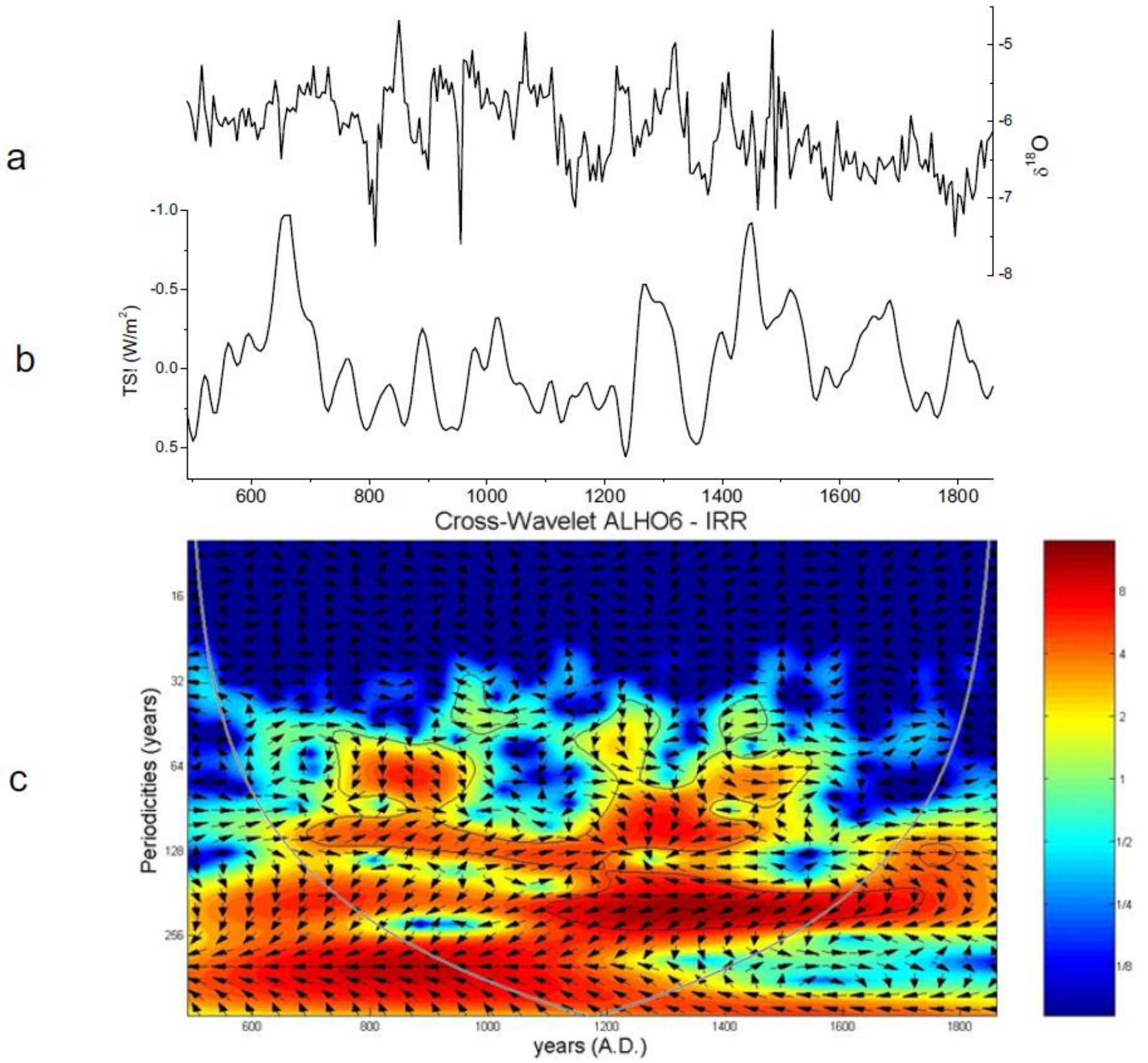

Supplementary Figure S9 | Cross-wavelet analysis (c) between ALHO6 $\delta^{18}O$ record (a) and reconstruction of total solar irradiance (b)[3]. The direction of the arrows to the left (as occurs during the periodicity centered around 208 years) is indicative of anti-phasing between the two time series. Analyses were perfomed using Matlab routines from Grinsted et al.[4].

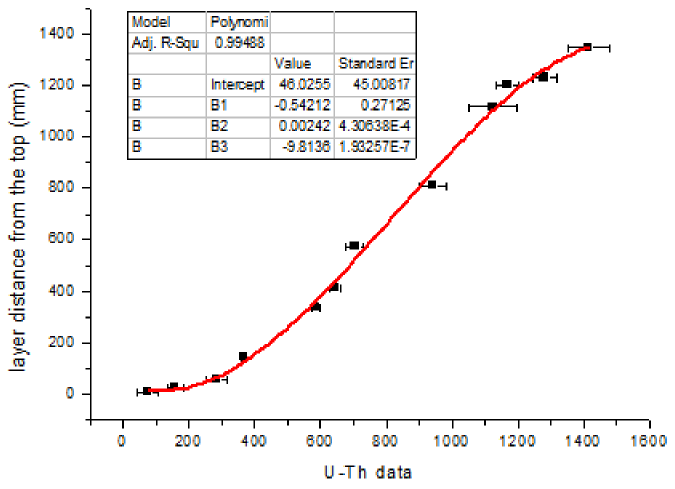

Supplementary Figure S10 | Polynomial fit between the distance of sampled layers (in mm) from the top of the stalagmite ALHO6 and U-Th age of layers.

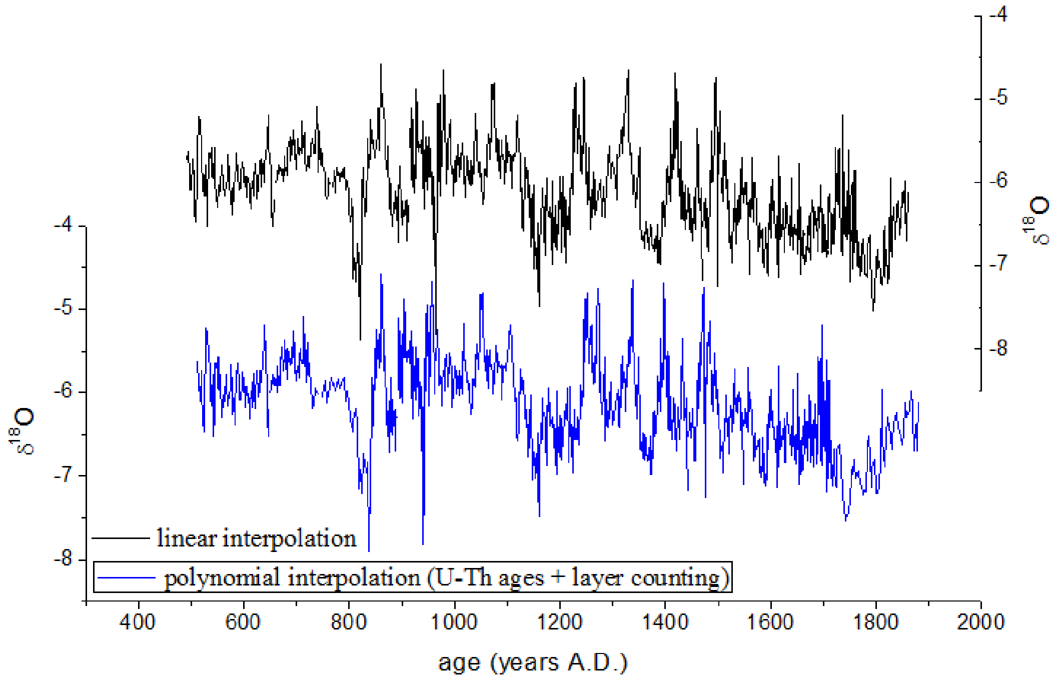

Supplementary Figure S11 | $\delta^{18}O$ from ALHO6 speleothem record based on linear interpolation between the U-Th ages (black) and based on polynomial interpolation (blue) derived from fit between U-Th ages and layer distance from the top as shown in Fig. S10.

Supplementary Table S1

**Sample Number**	**^{238}U (ppb)**		**^{232}Th (ppt)**		**d^{234}U* (measured)**		**^{230}Th / ^{238}U (activity)**		**^{230}Th Age (yr) (uncorrected)**		**^{230}Th Age (yr) (corrected)**		**d$^{234}U_{Initial}$** (corrected)**	
**Stalagmite ALHO6**														
**ALHO6-4**	3206	± 13	10287	± 40	644.6	± 3	0.00199	± 0.00015	132	± 10	**75**	**± 30**	644.7	± 3
**ALHO6-12**	5336	±15	10317	±208	676	±3	0,0029	±0,0000	192	±1	**158**	**±24**	676	±3
**ALHO6-22**	3197	±9	8900	±180	665	±3	0,0051	±0,0000	332	±2	**284**	**±34**	665	±3
**ALHO6-21**	3848	±10	2125	±43	670	±3	0.0058	±0.0000	378	±2	**368**	**±7**	670	±3
**ALHO6-78**	3856	±8	3544	±71	667	±2	0.0092	±0.0000	602	±2	**586**	**±12**	668	±2
**ALHO6-91**	6413	±18	8156	±164	692	±3	0,0103	±0,0000	667	±3	**645**	**±16**	694	±3
**ALHO6-109**	24042	± 116	66334	± 209	694	± 4	0,01164	± 0,001	751	± 14	**704**	**± 27**	695	± 4
**ALHO6-139**	4388	±12	17805	±359	655	±3	0,0153	±0,0001	1012	±5	**941**	**±51**	657	±3
**ALHO6-159**	3683	±11	21570	±436	661	±3	0.0186	±0.0001	1227	±6	**1,124**	**±73**	663	±3
**ALHO6-171**	3631	±12	10044	±203	691	±4	0.0187	±0.0001	1215	±6	**1,167**	**±34**	694	±4
**ALHO6-196**	3585	±13	10282	±208	644	±4	0.0200	±0.0001	1331	±7	**1,280**	**±37**	647	±3
**ALHO6-223**	4136	±17	20448	±417	630	±4	0.0223	±0.0001	1502	±10	**1,414**	**±63**	633	±4
**Stalagmite CUR4**														
**CUR4-3**	3550	±12	2591	±53	31	±2	0.0014	±0.0001	52	±1	**45**	**±5**	1890	±6
**CUR4-13**	2893	±7	1241	±25	77	±2	0.0020	±0.0001	78	±1	**73**	**±3**	1825	±4
**CUR4-32**	2654	±6	638	±13	220	±5	0.0032	±0.0001	123	±1	**120**	**±2**	1851	±4
**CUR4-48**	3171	±9	1792	±36	135	±3	0.0046	±0.0001	191	±1	**185**	**±5**	1644	±4

Supplementary Table S1: U-Th ages from stalagmites ALHO6 and CUR4. Analytical errors are 2σ of the mean.

$^{\alpha}\delta^{234}U = ([^{234}U/^{238}U]activity - 1) \times 1000$.

$^{\beta}\delta^{234}$Uinitial corrected was calculated based on ^{230}Th age (T), i.e., δ^{234}Uinitial = δ^{234}Umeasured X $e^{\lambda234*T}$, and T is corrected age.

$^{c}[^{230}Th/^{238}U]activity = 1 – e^{-\lambda230T} + (\delta^{234}U_{measured}/1000)[\lambda230/(\lambda_{230} - \lambda_{234})](1 - e^{-(\lambda230 - -\lambda234) T})$, where T is the age.

Decay constants are 9.1577×10^{-6} yr^{-1} for ^{230}Th, 2.8263×10^{-6} yr^{-1} for ^{234}U, and 1.55125×10^{-10} yr^{-1} for ^{238}U. Values from Cheng et al.[5].

[d] The degree of detrital ^{230}Th contamination is indicated by the [$^{230}Th/^{232}Th$] atomic ratio instead of the activity ratio.

[e]Age corrections were calculated using an average crustal $^{230}Th/^{232}Th$ atomic ratio of $4.4 \times 10^{-6} \pm 2.2 \times 10^{-6}$.

Those are the values for a material at secular equilibrium, with the crustal $^{232}Th/^{238}U$ value of 3.8. The errors are arbitrarily assumed to be 50%[2].

	Pau d'Alho cave		Curupira cave	
**date**	**hall of stalagmites**	**entrance**	**hall of stalagmites**	**entrance**
**Feb (2013)**	X	530 ppm	992 ppm	1200 ppm
**Jul (2013)**	1132 ppm	436 ppm	520 ppm	510 ppm
**Mar (2014)**	7449 ppm	456 ppm	2353 ppm	933 ppm
**Mar (2016)**	9072 ppm	492 ppm	11042 ppm	3200 ppm

Supplementary Table S2 | CO_2 measured in the caves Pau d'Alho and Curupira.

Supplementary References

1. Schneider, U, et al. GPCC's new land surface precipitation climatology based on quality-controlled in situ data and its role in quantifying the global water cycle. *Theor. Appl. Climatol.*, **115**, 14-40, 2014.

2. Hammer, Ø., Harper, D. A. T. & Ryan, P. D. PAST: Paleontological Statistics software package for education and data analysis. *Paleonto. Elect*., **4**, 1-9 (2001).

3. Steinhilber, F., Beer, J. & Fröhlich. C. Total solar irradiance during the Holocene. *Geophys. Res. Lett*., **36**, L19704 (2009).

4. Grinsted, A., Jevrejeva, S. & Moore, J. Application of the cross wavelet transform and wavelet coherence to geophysical time series, *Nonlinear Proc. Geoph*., **11**, 561–566 (2004).

5. Cheng, H. et al. The half-lives of uranium-234 and thorium-230. *Chem. Geol*., **169**, 17-33 (2000).
